# Protective action of ultrasound-guided electrolysis technique on the muscle damage induced by notexin in rats

**DOI:** 10.1371/journal.pone.0276634

**Published:** 2022-11-28

**Authors:** Adrian Jorda, Juan Campos-Campos, Constanza Aldasoro, Carlos Colmena, Martin Aldasoro, Kenia Alvarez, Soraya L. Valles

**Affiliations:** 1 Department of Physiology, School of Medicine, University of Valencia, Valencia, Spain; 2 Faculty of Nursing and Podiatry, Department of Nursing, University of Valencia, Valencia, Spain; University of Minnesota Medical School, UNITED STATES

## Abstract

It is known that exercise can be one of the causes of muscular damage. In recent times, physiotherapists and medical professionals have been employing USGET techniques to stimulate muscle recovery to improve its performance after the injury. We pretend to analyse if the Ultrasound-guided electrolysis (USGET) technique could reduce muscle damage, inflammation, and pain in the present study. Female Wistar rats were assigned to one of three different groups: control (C), notexin (NOT) and notexin with USGET (electrolysis at 6mA) (NOT+USGET). We used the USGT technique, based on electrical stimulation with a continuous current of 4 pulses at an intensity of 6 mA for 5 seconds, conveyed to the muscle. The response was tested with motor function tests. In these tests, we could observe an increase in time and foot faults when crossing a beam in the NOT group compared to C group rats. On the other hand, a significant decrease in both variables was detected in the NOT+USGET compared to the NOT group. Muscle power was measured with a grip strength test, obtaining far better performances in NOT+USGET rats when compared to NOT rats. Moreover, the USGET technique prevented the increase of pro-inflammatory proteins IL-6 and chemokines CCL3 (Chemokine (C-C motif) ligand 3), CCL4 (Chemokine (C-C motif) ligand 4), and CCL5 (Chemokine (C-C motif) ligand 5) with their receptor CCR5 (C-C chemokine receptor type 5), induced by notexin in the quadriceps. At the same time, the study evidenced a decrease in both CCR8 (C-C chemokine receptor type 5,) and NF-ᴋB (nuclear factor- ᴋB) expressions after USGET treatment. On the other hand, we obtained evidence that demonstrated anti-inflammatory properties of the USGET technique, thus being the increase in IL-10 (Interleukin 10) and IL-13 (Interleukin 13) in the NOT+USGET group compared to the NOT group. Furthermore, when applying NSGET after damage, an increase in anti-inflammatory mediators and reduction of pro-inflammatory mediators, which, overall, promoted muscle regeneration, was observed. These results support the idea that the NSGET technique improves muscle recovery after toxic damages, which would justify its employment.

## Introduction

Homeostasis is an essential mechanism that must be preserved in all organic systems, including skeletal muscle since it plays a magnanimous role in maintaining their integrity. Skeletal muscle, which we will study in the present research, undergoes continuous dynamical changes in response to injuries, ageing processes, pathologies, and physical exercise per se [1]. A common underlying feature for all these processes of them is inflammation. The inflammatory response has two main phases, the pro-inflammatory phase, with infiltration of leukocytes and the anti-inflammatory phase, where skeletal muscle regenerates [2,3]. Recovery of damaged tissue is primarily controlled by macrophages, which orchestrate the inflammatory response [4–6]. Cytokines, chemokines, pro-and anti-inflammatory proteins, leukocytes, and phagocytes act in the inflammatory process after muscle injury [4,7].

There are two main inflammatory processes: acute and chronic inflammation. Acute inflammation has a fast onset and an equally fast resolution. However, chronic inflammation does not achieve complete recovery, with macrophages and lymphocytes being the primary cells involved [8,9]. Both are responsible for producing cytokines and chemokines as the primary chemical mediators of this type of inflammation. In fact, in chronic inflammation and contractile dysfunction, IL-1β (Interleukin 1β) and TNFα (Tumour Necrosis Factor α) play a crucial role [10]. In addition, ROS (Reactive Oxygen Species) derived from muscle and NO (Nitric Oxide) play a part in force decrement, causing muscle atrophy [11]. On the other hand, IL-6 (Interleukin 6) is a critical inflammatory cytokine in the inflammatory process that facilitates muscle atrophy by decreasing its anabolism [1]. If the agent causing the inflammation cannot be removed, or if there is any interference with the healing process, an acute inflammatory response can progress to the chronic stage. Repeated episodes of acute inflammation can also lead to chronic inflammation. The response to sudden body damage is to send inflammatory cells to the injury. These cells start the healing process. In chronic inflammation, the body continues to send inflammatory cells even when there is no danger from the outside. Techniques that can reduce or eliminate chronic inflammation by repairing the damaged area in the future should be a priority in bodily injury research [8,9].

Cytokines and chemokines are small proteins released by our cells that act as messengers between the different cells of the immune system. They are responsible for coordinating an effective immune response according to infection and regulating inflammation. They could activate, attract, and direct diverse families of circulating leukocytes to damaged sites [12,13]. Cytokines and Chemokines not only participate in the coordination of leukocyte movement in inflammatory processes but are also important in multiple physiological and pathological processes: development of the immune system; surveillance, memory, immune response, and regulation; inflammation; embryogenesis, angiogenesis, and organogenesis; nervous system development and function; germ cell migration; cancer development and metastasis [12,13].

Coordination between inflammatory and regenerative processes would be necessary to reduce the inflammatory response to increase functional muscle recovery. In recent years, physical therapists and medical specialists have used Ultrasound-guided electrolysis (USGET). This technique produces a non-thermal electrolytic ablation that induces a controlled inflammatory response, allowing the activation of cellular mechanisms involved in phagocytosis and regeneration of damaged soft tissue [14,15]. USGET technique is a recovery method to improve performance. The technique uses a galvanic current to produce a chemical reaction, which causes the dissociation of sodium chloride and water molecules [15–17]. Moreover, it facilitates wound healing in the patellar tendon and damaged muscle in rats [16,17]. Furthermore, the main and most current accepted theory is that galvanic current has been found to activate the inflammasome to promote the production of type I collagen in the tendon. [14]. Nonetheless, there are not many studies on the action of the USGET technique at a biochemical and physiological level that can explain the effects previously indicated. Therefore, this study aimed to explore the effects of the USGET technique on muscle damage and inflammation. Our experimental study aimed at seven days of Notexin-induced injury, determining the action of the USGET technique in recovering from muscle damage. Notexin (from snake venom), a pre-synaptic phospholipase A2 neurotoxin, is known to induce both destruction and regeneration of skeletal muscle fibres, giving a reliable model of skeletal muscle injury-regeneration in rat [7,18–20] and mouse [21]. Although notexine has neurotoxic effects due to its presynaptic action, myotoxic effects and myoglobinuria have also been described. In addition, it can act as a procoagulant causing prothrombin cleavage. This procoagulant action precedes the muscular toxic effects [22–24]. Furthermore, we tested if the application of USGET after notexin-induced muscle damage could act as a defensive technique on rat muscle. Another aspect studied was the inflammatory state and the possible recovery in exercise performance after applying the USGET technique.

## Material and methods

### Notexin administration and USGET technique

Twenty female Wistar rats (weight 200–250 g; age 7 months) (a minimum of 20 rats were necessary for environment and protein expression measurement) were randomly divided into 3 groups with 5 in each group, including: control (C), notexin (NOT), and notexin with USGET (6 mA) (NOT+USGET). The rats (with and without notexin) were fed ad libitum on a standard diet (Letica, Barcelona, Spain) and were kept on a 12-h light/12-h dark cycle with room temperature maintained at 22ºC. When notexin was used, only one leg was injected (to prevent a double notexin injection). The application of notexin, by intramuscular injection, was carried out in the central zone of the quadriceps muscle. 200 μl of notexin were injected intramuscularly at 10 μg/ml in the quadriceps to produce muscle injury. Notexin was injected 7 days before USGET application. The rats in control group were injected with 200 μl of saline solution.

The treatment with USGET was carried out during the following 7 days after notexin administration and applied once on the 7^th^ day and once on the 11^th^ day. These days are applied since sufficient days must be left for the notexin to act chronically at the muscular level. The application of electrolysis on day 7 will give an idea of the fast action and 11 days of its later action. Before the injection of notexin and USGET treatment, rats were anesthetized via inhalation (5% inofluoran by induction and 2% maintenance). The application of the USGET was carried out in the same area where the notexin was applied and consisted in the administration of a continuous current of 4 pulses at an intensity of 6 mA for 5 seconds conveyed to the muscle. An acupuncture needle with a 0.32 mm diameter was used as electrode. In experiments not included in the manuscript, different intensities were used (2, 3, 6 and 8 mA), showing that the most suitable was 6 and 8 mA. The seconds were also varied (2, 5, 10), highlighting that it was best to use 5 seconds. Animals were euthanized at the end of the study (pentobarbital 50 mg/Kg) and quadriceps muscle was removed (14^th^ day after notexin administration) and the central area was used to biochemistry determination. All animal procedures were carried out in accordance with the European legislation on the use and care of laboratory animals (EEC 86/609).

### Balance beam test

This test was used to assess sensorimotor function and balance in rats. The animals had to pass through a narrow beam (1 x 100 cm) elevated 1’5 m above the floor, reaching a dark box at the end of it. To evaluate the rats’ ability in this test, they were forced by illuminating the beginning of the beam with white light. The time required to reach the escape box at the end of the beam and the number of forelimb and hind-limb paw slips were both recorded. Paw slips are defined as any paw coming off the beam’s top or side. The same day that the test was affected, four trials were performed to familiarize the rats with the beam and the dark box at the end of it. After each trial, the beam and box were cleaned with ethanol.

### Grip strength

Grip strength was measured non-invasively by taking advantage of the rat’s instinct to hold on while being pulled backwards gently. This method can evaluate limb grip using a 0.1 cm diameter horizontal bar attached to a force transducer. The time interval the rat was able to hold the bar was recorded, and the latency of grip loss was considered an indirect measure of grip strength [25], holding a maximum cut-off time of 60 seconds. The advantages are that grip strength is reproducible and can be measured repeatedly in rats to detect progressive motor deficits, therapy value and genetic modifications.

### Western-blot analysis

Protein extracts from quadriceps were mixed with equal volumes of SDS buffer (0.125 M Tris-HCl, pH 6.8, 2% SDS, 0.5% (v/v) 2-mercaptoethanol, 1% protease inhibitors, 1% bromophenol blue and 19% glycerol) and then boiled for five minutes. Protein concentration was determined using a modified Lowry method [26]. Proteins were separated using SDS-PAGE gels and transferred to polyvinylidene difluoride membranes. These membranes were previously impregnated with methanol in a humid environment using a transfer buffer (25 mM Tris, 190 mM glycine and 20% methanol). Membranes were blocked with 5% BSA in TBS containing 0.05% Tween-20 and then incubated overnight with the corresponding antibodies following the manufacturer’s recommendations. The blots were washed three times with a washing buffer (phosphate-buffered saline, 0.2% Tween 20) for 5 min each and then incubated for one hour with a secondary horseradish peroxidase-linked anti-mouse IgG antibody (Cell Signalling Technologies, Barcelona, Spain). As mentioned above, the blots were washed three times and developed using the enhanced chemiluminescence (ECL) procedure as specified by the manufacturer (Amersham Biosciences, Barcelona, Spain). Auto-radiographic signals were assessed using ImageJ software (NIH Image, National Institutes of Health, Bethesda, MD, USA). The antibodies: monoclonal anti-CCR5 (1:500), monoclonal anti-CCR8 (1:500), monoclonal anti-NFᴋB (1:500), and monoclonal anti-IᴋB (1:500) antibodies were from Abcam Biotechnology, and the monoclonal α-tubulin (1:1000) from Santa Cruz Biotechnology (Madrid, Spain).

### Real-time polymerase chain reaction analyses

Quadriceps samples were collected from each rat into an RNAlater solution (Ambion, Austin, TX, USA), an RNA stabilization reagent, following the manufacturer’s instructions. Total RNA was extracted with a Tripure isolation reagent (Roche Molecular Biochemical, Basel, Switzerland). Its concentration and integrity were assessed in RNA 6000 Nano Labchips using Agilent 2100 Bio-analyzer (Agilent Technologies, Foster City, CA, USA). Ready-to-use primers and probes from the assay-on-demand service of Applied Biosystems were used for the quantification of selected target gene: CCL3 (Mm00441259_g1), CCL4 (Mm00443111_m1), CCL5 (Mm01302428_m1), CCR5 (Mm01963251_s1), CCL1 (Mm99999220_Mh), CCR8 (Mm99999115_s1) and endogenous reference gene β-actin (Mm00607939_s1). RNA samples were reverse transcribed using random hexamers and MultiScribe reverse transcriptase (Applied Biosystems). After complementary DNA synthesis, real-time polymerase chain reaction (RT-PCR) was carried out using the ABI Prism 7900HT Sequence Detection System (Applied Biosystems). Samples were run in triplicate, and expression changes were generated by calculating 2^-ΔΔCt^ [27]

### Determination of cytokines IL-6, IL-13, and IL-10

Plasma was obtained from the rats and used to determine IL-16, IL-13, and IL-10 cytokine concentrations (pg/ml) using ELISA kits (Pierce Biotechnology, Inc.). The blood was obtained from the lateral vein of the tail (1 ml) post anaesthesia. First, plasma was obtained, using afterwards 100 μl of it for each test.

### Data analysis and statistics

All values are expressed as means ± SD. The differences between rat groups were determined with unpaired Student’s t-test. All statistical analyses were performed using Graph-Pad Prism software (GraphPad Software Inc., San Diego, CA, USA). The data were analyzed by parametric ANOVA (the variances are homogeneous). In addition to this, the Scheffe method was used to determine post-hoc pair-wise comparisons. Statistical significance was accepted at a p-value < 0.05.

## Results

### Motor function

**Fig 1:**
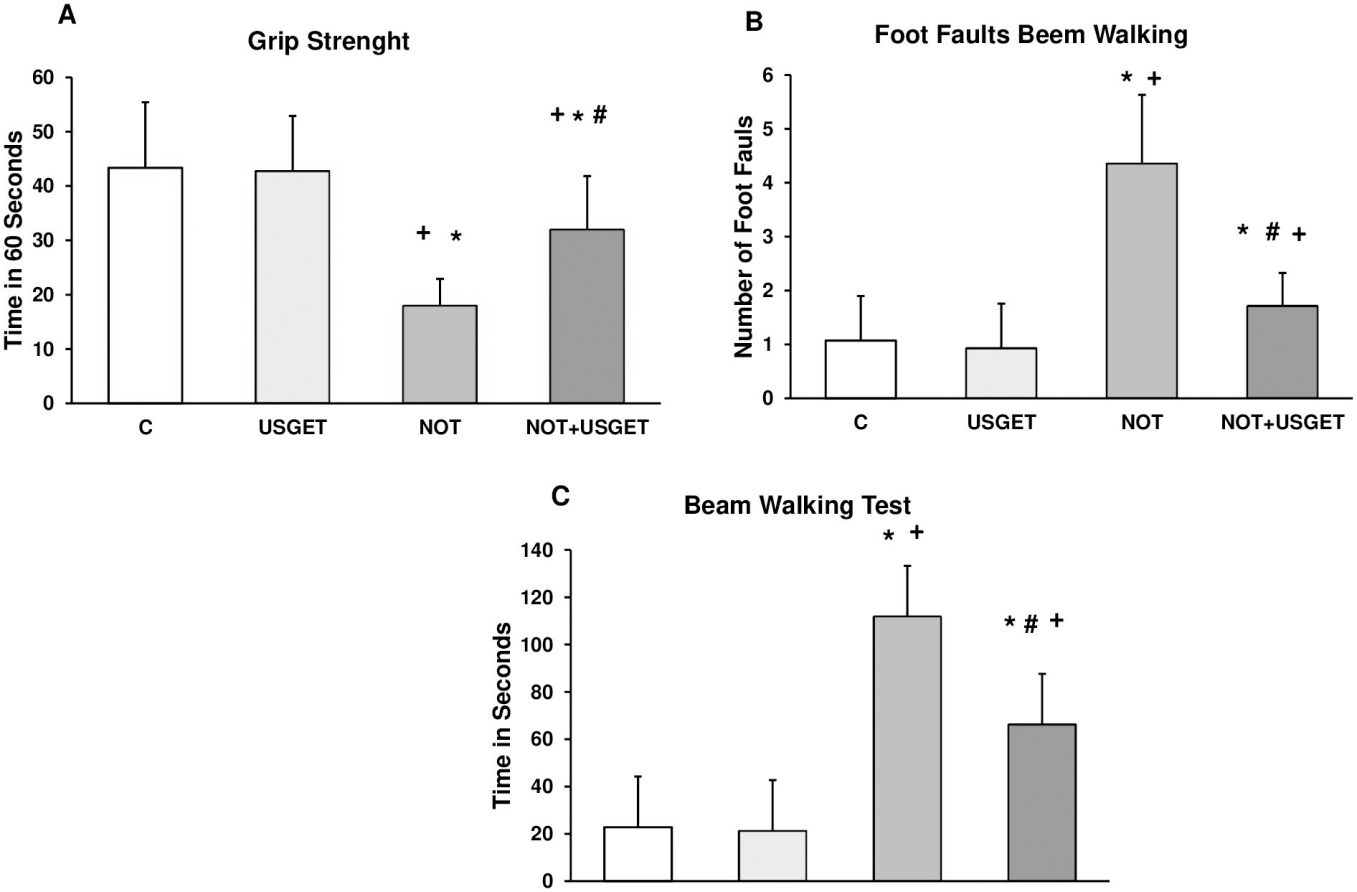
Rats were tested without notexin (control, C or USGTE (6 mA)) or with notexin (NOT) or notexin with USGTE (6 mA) (NOT+USGTE). A: Grip Strength (time in 60 seconds). B: number of foot faults. C: Time to cross the beam (sec). *p<0.01 vs. control. #p<0.01 vs. notexin.

The rat’s ability to pass through a narrow beam showed the difference of activity between control (C), notexin (NOT) and notexin+USGET (NOT+USGET) rats using the beam-walking test. Grip strength test showed that NOT rats presented a significant time reduction compared to C rats. In NOT+USGET rats, a significant time increase was detected compared with the NOT group (Fig 1A). In addition, the number of foot fouls was higher in the NOT group compared with C and NOT+USGET group (Fig 1B). The ability to complete the beam-walking test is shown in Fig 1C. The NOT group spent more time completing this test than the C group. The use of the USGET technique resulted in a reduction of time to complete the test compared with the NOT group (Fig 1C).

### Expression of IL-6, IL-13, and IL-10 cytokines

**Fig 2:**
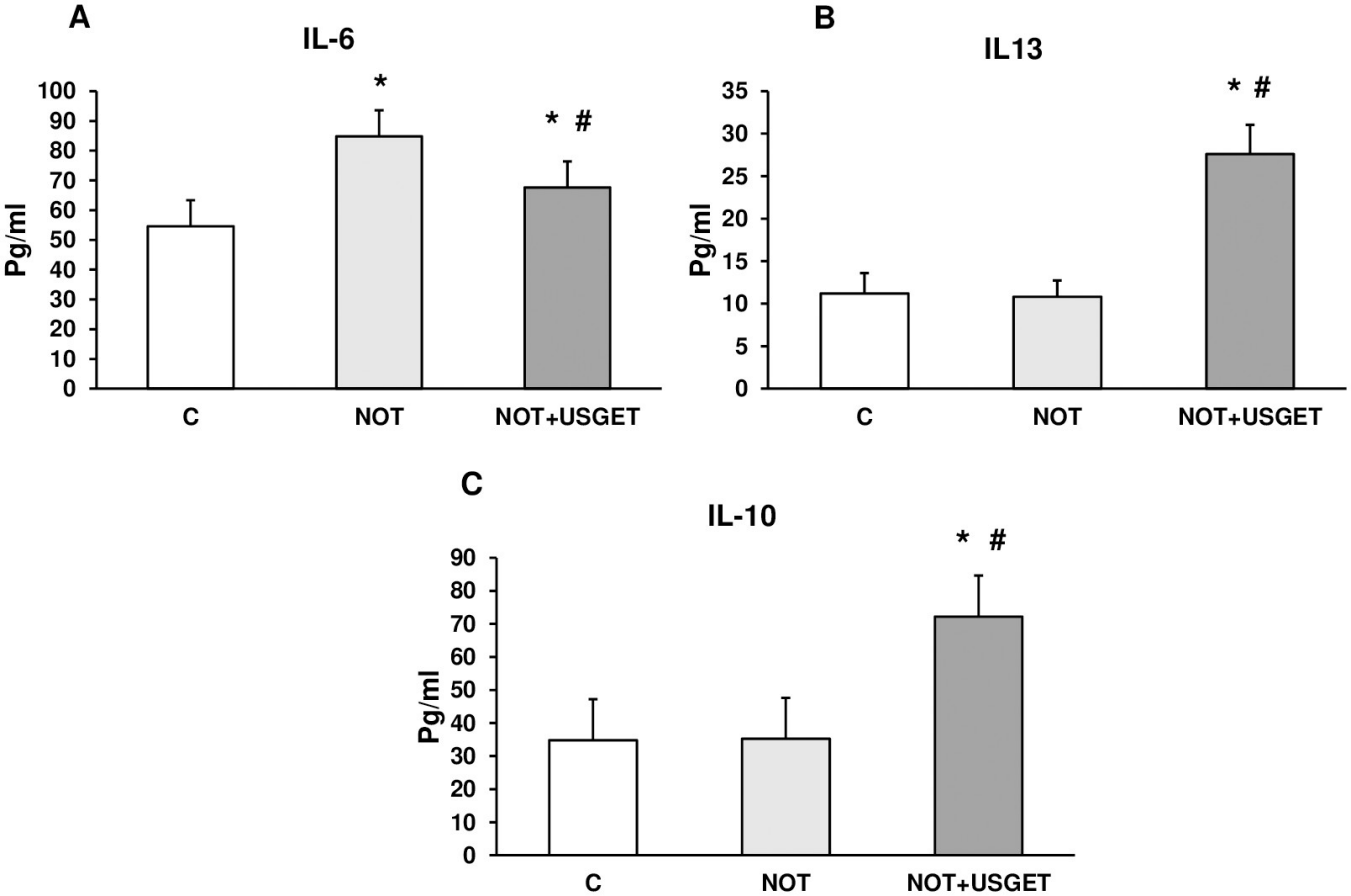
Plasma from rats without notexin (C) or notexin (NOT) or notexin with USGTE (6 mA) (NOT+USGTE) was collected. IL-6 (A), IL-13 (B), and IL-10 (C) were determined by ELISA. Values are means ± SD from five independent experiments. *p<0.03 vs. control. #p<0.03 vs. notexin.

Serum from the rats (C, NOT and NOT+USGET) was assayed to determine the cytokines by ELISA. Fig 2A shows that NOT induced a significant increase in IL-6 pro-inflammatory cytokine compared to C rats. Conversely, in NOT+USGET rats, a significant reduction was detected compared with the NOT group. Under our experiments, neither anti-inflammatory cytokines IL-13 nor IL-10 expressed significant differences between C and NOT groups. On the contrary, NOT+USGET showed a significant increase compared with C and NOT groups (Fig 2).

### Expression of CCL3, CCL4 and CCL5 chemokines

**Fig 3:**
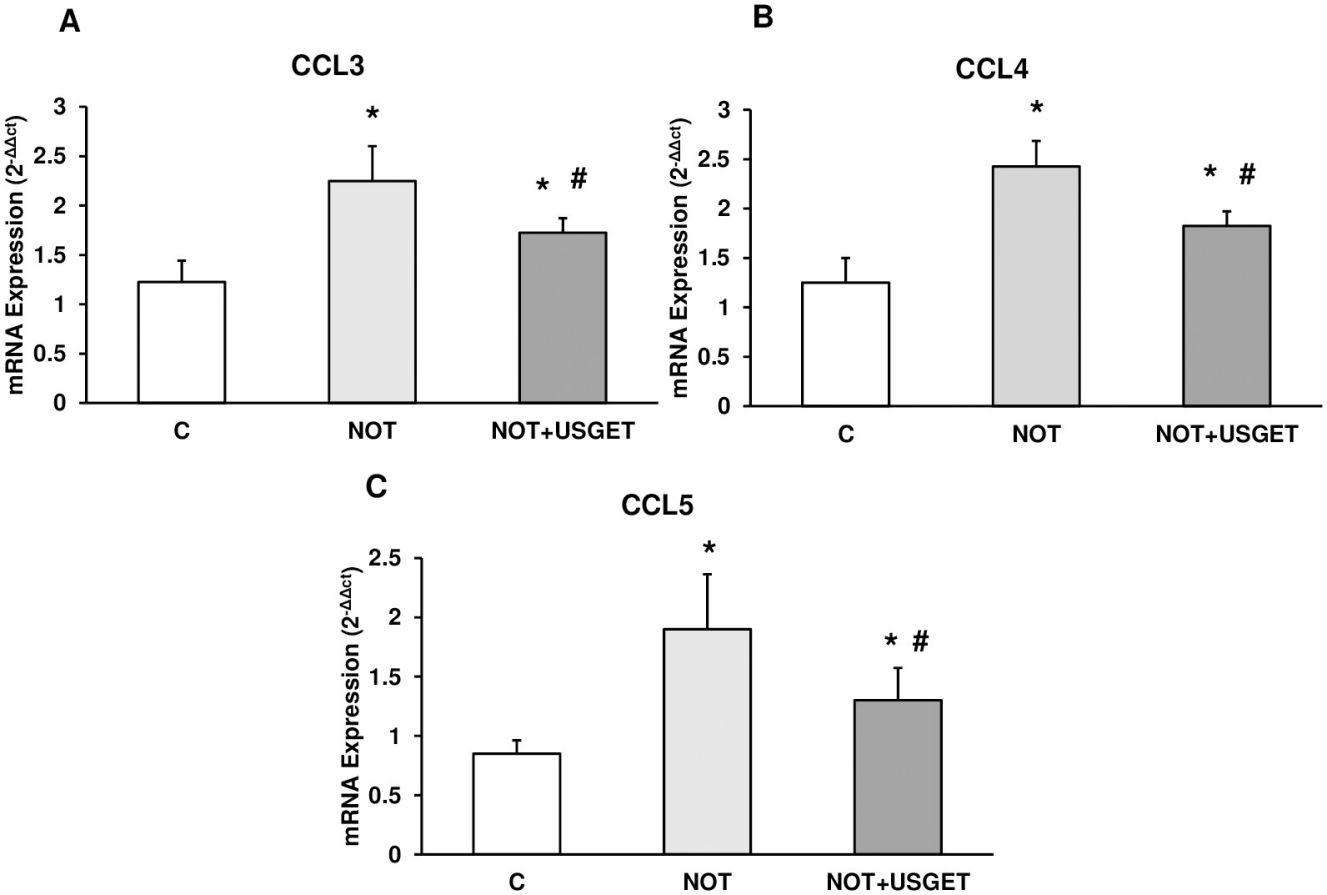
Rat muscle without notexin (C) or with notexin (NOT) or notexin with USGTE (6 mA) (NOT+USGTE) was collected to determine A: CCL3, B: CCL4, and C: CCL5 mRNA chemokine expression. Values are means ± SD from five independent experiments. *p<0.05 vs. control. #p<0.05 vs. notexin.

To determine the expression of CCL3, CCL4 and CCL5 pro-inflammatory chemokines, we measured mRNA expression by real-time RT-PCR in quadriceps from rats. The expression in all chemokines analyzed was higher in the NOT group compared to the C group. In addition, chemokine expression (CCL3, CCL4 and CCL5) significantly decreased in the NOT+USGET group compared to the NOT group (Fig 3).

### Protein expression of the CCR5 receptor

**Fig 4:**
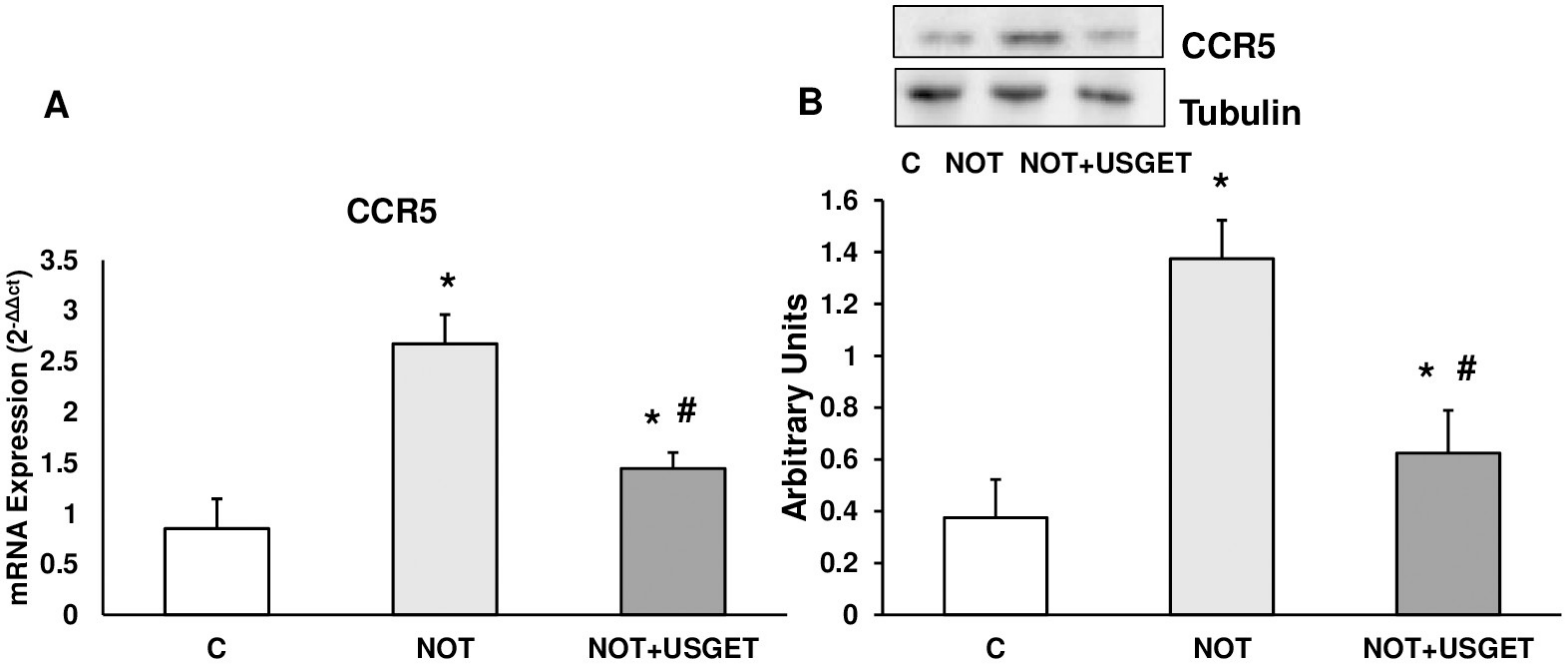
Rat muscle without notexin (C) or with notexin (NOT) or notexin with USGTE (6 mA) (NOT+USGTE) was collected to determine A: CCR5 mRNA expression and B: CCR5 protein expression by Wester-blot. A representative immunoblot is shown in the panel. CCR5 and Tubulin cropped from different parts of the different gels. Values are means ± SD from five independent experiments. *p<0.04 vs. control. #p<0.04 vs. notexin.

CCR5 is the receptor of CCL3, CCL4 and CCL5 chemokines. Western-blot and RT-PCR techniques were used to determine the expression of this receptor. Fig 4 shows that CCR5 expression significantly increased in NOT and NOT+USGET groups compared to the C group. Moreover, CCR5 expression significantly diminished in the NOT+USGET group compared to the NOT group (Fig 4).

### Protein expression of CCL1 chemokines and its CCR8 receptor

**Fig 5:**
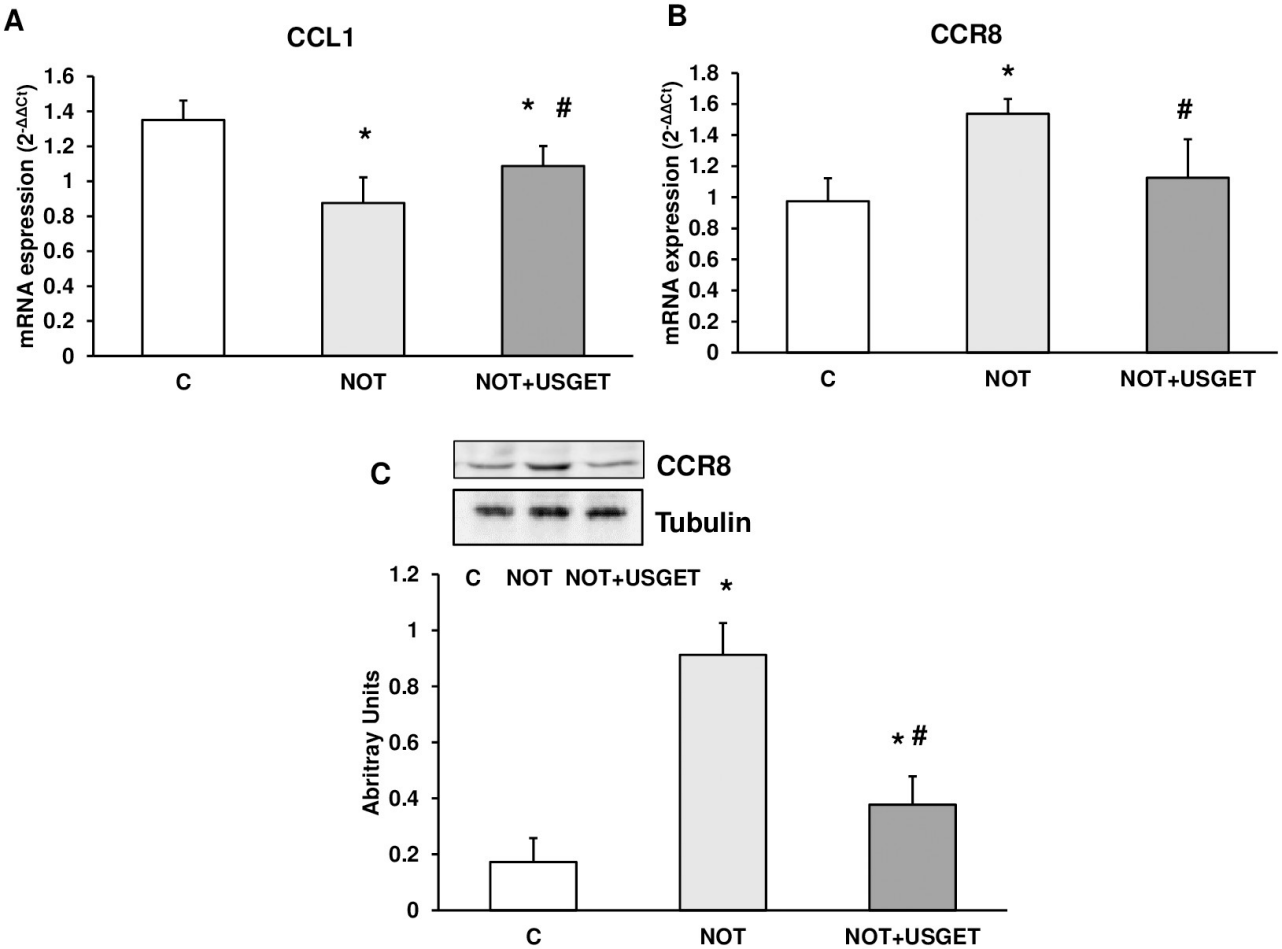
Rat muscle without notexin (C) or with notexin (NOT) or notexin with USGTE (6 mA) (NOT+USGTE) was collected to determine A: CCL1 and B: CCR8 by mRNA expression, and C: CCR8 by Western-blot. CCR8 and Tubulin cropped from different parts of the different gels. Data are means ± SD from five independent experiments. *p<0.04 vs. control. #p<0.04 vs. notexin.

To determine the expression of CCL1 chemokine, we measured mRNA expression by real-time RT-PCR. NOT group presented a significantly reduced expression of CCL1 in comparison with the C group. Furthermore, the NOT+USGET group shows an increment of this chemokine concerning the NOT group. CCR8 is the receptor of CCL1 chemokine. Western-blot and RT-PCR techniques were used to determine the expression of this receptor. Fig 5 shows that CCR8 expression was significantly increased in NOT and NOT+USGET groups compared to C rats. Furthermore, CCR8 expression significantly diminished in the NOT+USGET group compared to the NOT group (Fig 5).

### Protein expression of NFᴋB and IᴋB

**Fig 6:**
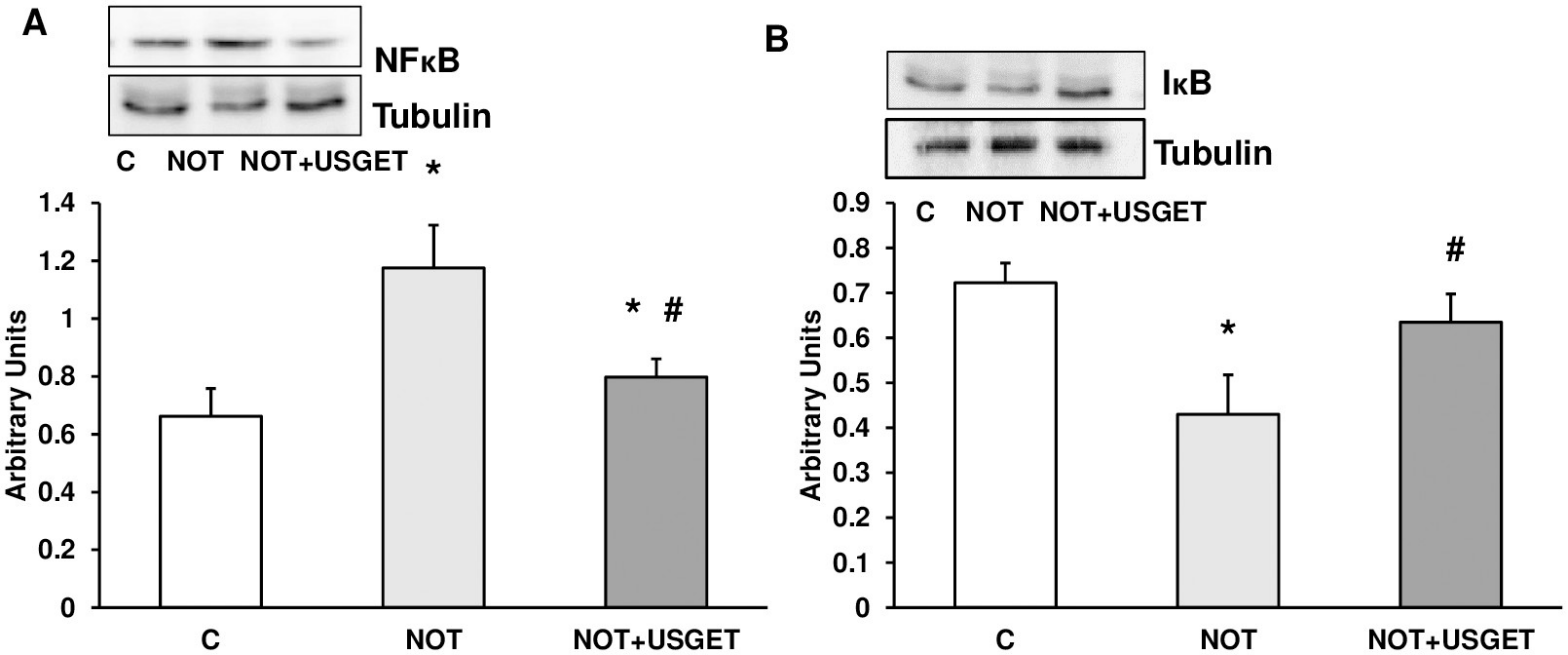
Rat muscle without notexin (C) or with notexin (NOT) or notexin with USGTE (6 mA) (NOT+USGTE) was collected to determine A: NFᴋB and B: IᴋB by Western-blot. NFᴋB, IᴋB and tubulin cropped from different parts of the different gels. Data are means ± SD from five independent experiments. *p<0.05 vs. control. #p<0.05 vs. notexin.

The expression of both proteins was tested using the Western-blot technique. Fig 6 shows that the transcription factor NFᴋB was significantly increased after notexin addition compared with the control group. On the contrary, in the NOT+USGET group, significant decrement was detected compared with the NOT group. Moreover, the NOT group presented a decrease in IᴋB protein compared with the control group. On the other hand, when USGET was used, a diminished expression was noted (Fig 6).

## Discussion

The present study evaluated the changes produced using the USGET technique on rats’ damaged muscle induced by notexin. It focuses on the USGET technique’s effect on muscle tissue showing biomolecular mechanism and motor functions. We hypothesize that this technique could protect the damaged muscle from further inflammation, reducing pro-inflammatory proteins and increasing anti-inflammatory proteins, favouring tissue regeneration, motor function, and exercise performance.

In the last years, it has been published that the application of USGET in humans does not show adverse effects on sports performance [28,29], and the technique can be used to improve recovery after muscle damage [15]. Animal studies demonstrated that percutaneous electrolysis applied in an experimentally induced model of Achilles tendinopathy could increase the expression of some genes associated with collagen regeneration and extracellular matrix remodelling [30]. In a human study, high-intensity percutaneous electrolysis was applied (3 shifts of 3 mA × 3 s) and its effects on pain were determined [31], showing that strong electroacupuncture can induce greater analgesic effects than weak electroacupuncture (0.3 mA × 90 s). Supporting our experimental conditions, the protocol that used 3 mA for three seconds and three repetitions in rats was the most effective than other protocols with less mA and less time [32]. In our study (6 mA × 5 sec), after administration of notexin, we noted that USGET treatment reverses inflammatory processes. USGET diminished the expression of pro-inflammatory cytokine (IL-6), chemokines (CCL3, CCL4 and CCL5), and CCR5 and CCR8 receptor expression after notexin addition. Furthermore, USGET treatment decreased transcription factor NF-ᴋB compared with the notexin group. Moreover, an increase of anti-inflammatory mediators such as IL-13 and IL-10 were detected.

The entry of inflammatory cells after muscle injury favours regeneration through the absorption and digestion of necrotic cells and the elimination of waste [33]. In addition, inflammatory cells can produce cytokines, chemokines, and growth factors involved in the muscle damage process and sometimes in posterior regeneration [10,34]. In an early stage of muscle regeneration, inflammation of the damaged muscle can aid regeneration. However, a continuous or excessive inflammation can lead to muscle fibrosis [35,36]. Using techniques to reduce inflammation and its prolongation over time could lead to more significant functional recovery from the injury. In melanoma, neuroblastoma cells and muscle tissue, deleterious notexin action was demonstrated [7,37]. In decreased myogenic expression and subsequent myotube formation, changes in cytokine expression are the most likely explanation. Potentially mitogenic substances for myoblasts are driven by IL-6, IL-1, and TNF-α pro-inflammatory mediators [38,39]. It has also been published that these pro-inflammatory mediators act as myogenic differentiation inhibitors [40].

Furthermore, the addition of IL-6 cytokine to myoblasts in culture increases their proliferation but not cell fusion [41]. The genetic ablation of IL-6 slows muscle growth in a model of compensatory muscle hypertrophy. The gastrocnemius tendon was sectioned to rise packing and growth in the synergistic plantaris muscle in these mice. Furthermore, these authors demonstrate that ablation of IL-6 diminishes muscle growth [42,43]. The decrement in IL-6 expression by USGET treatment will improve muscle regeneration and functional muscle recovery by reducing the inflammatory response. However, the contradiction between our results and the data exposed before is apparent. USGET technique produces, in the beginning, destruction of tissue by cytochrome c and SMAC/diablo [16,17] to eliminate destroyed cells by the injury. However, after a time, USGET contributed to recovery the of muscle damage.

M1 macrophages are pro-inflammatory and responsible for inflammatory signalling, while M2 are anti-inflammatory macrophages that participate in the resolution of the inflammatory process and produce anti-inflammatory cytokines, thereby contributing to tissue healing [44]. M2 macrophages arrive at their peak concentration in muscle regeneration activated by anti-inflammatory cytokines, such as IL-10 and IL-13 [44,45]. Furthermore, IL-10 concentrations increase in muscle, decreasing myofiber injury, when regulatory T cells increase [46]. Our data showed up-regulation of IL-10 mRNA level in tibialis anterior injected with nitric oxide was detected on notexin-induced muscle damage [47].

Chemokines regulate the movement of leukocytes in response to injury and disease. They are small molecules that are liberated to activate the inflammation state. Furthermore, chemokines and their receptors are highly expressed after muscle injury [43,48]. In HIV (human immunodeficiency virus) infection, the role of CCR5 has been mainly studied [49]. This receptor is a major co-receptor for the entry of HIV into target cells [50,51]. Moreover, CCR5 is expressed in other tissues, such as in the CNS [52–54]. Myoblasts constitutively express the CCR5 receptor, and cell proliferation occurs when stimulated with chemokines, CCL3 and CCL4 [55]. An increment in CCL3, CCL4 and CCR5 in injured muscle was detected by different authors [56–58], as shown in our study after notexin addition. Using the USGET technique, we demonstrate a decrease in CCR5 and its chemokines (CCL3, CCL4 and CCL5), showing a possible muscle recovery produced by this technique.

CCL1 was detected after USGET treatment demonstrating muscle recuperation by controlling inflammation and perhaps improving its recovery after damage. Activated monocytes and lymphocytes secrete CCL1. This chemokine is the ligand of CCR8 and is a potent chemoattractant in this kind of cells [59,60]. Our experiments noted changes in CCR8 expression and its chemokine, CCL1, in skeletal muscle after notexin damage compared to the control group. We also detected changes using the PIE technique after notexin damage. We demonstrate an increase in CCL1 expression in NOT+USGET compared to the notexin group and a decrease in its receptor (CCR8) expression. This data indicates a reduction in CCR8 receptor expression to supply upregulation of its chemokine, CCL1.

Additionally, the increase in CCR8 expression after notexin damage contributes to pain development [61]. So, our data indicate that USGET treatment could produce a pain reduction in skeletal muscle damage. The combination of 1α,25-dihydroxyvitamin D3 and prostaglandin E2 (PGE2) produce a high CCR8 expression, showing the importance of tissue environments in maintaining cellular immune surveillance networks within different tissues [62]. However, our study has not analyzed muscle damage caused by physical exercise. Consequently, we cannot compare damage induced by notexin with damage induced by physical exercise.

The NF-κB is sequestered in the cytoplasm by IκB and keep it in an inactive state. On the other hand, degradation of IκB activates NF-κB [63,64]. In our results, after notexin addition a decrease of IκB and an increase in NF-κB were detected. The transcription factor NFᴋB is upregulated in inflammation conditions and regulates innate and adaptive immune functions [65]. This factor induces the expression of cytokines and chemokines participating in inflammasome regulation. In inflammatory situations, the activation of NFᴋB contributes to the pathogenic processes [66,67] as we detected after notexin injection. After the application of PIE technique, an increase in IκB expression occurs, sequestering NF-κB and inhibiting it, and preventing so the development of secondary inflammatory processes [65]. Therefore, developing therapeutic strategies based on NFᴋB inhibition would be the next step to diminish inflammation muscle damage. In this work, we demonstrate a decrease in NFᴋB protein expression in NOT+USGET compared with the notexin group, indicating the possible efficacy of this technique for that purpose.

**Fig 7:**
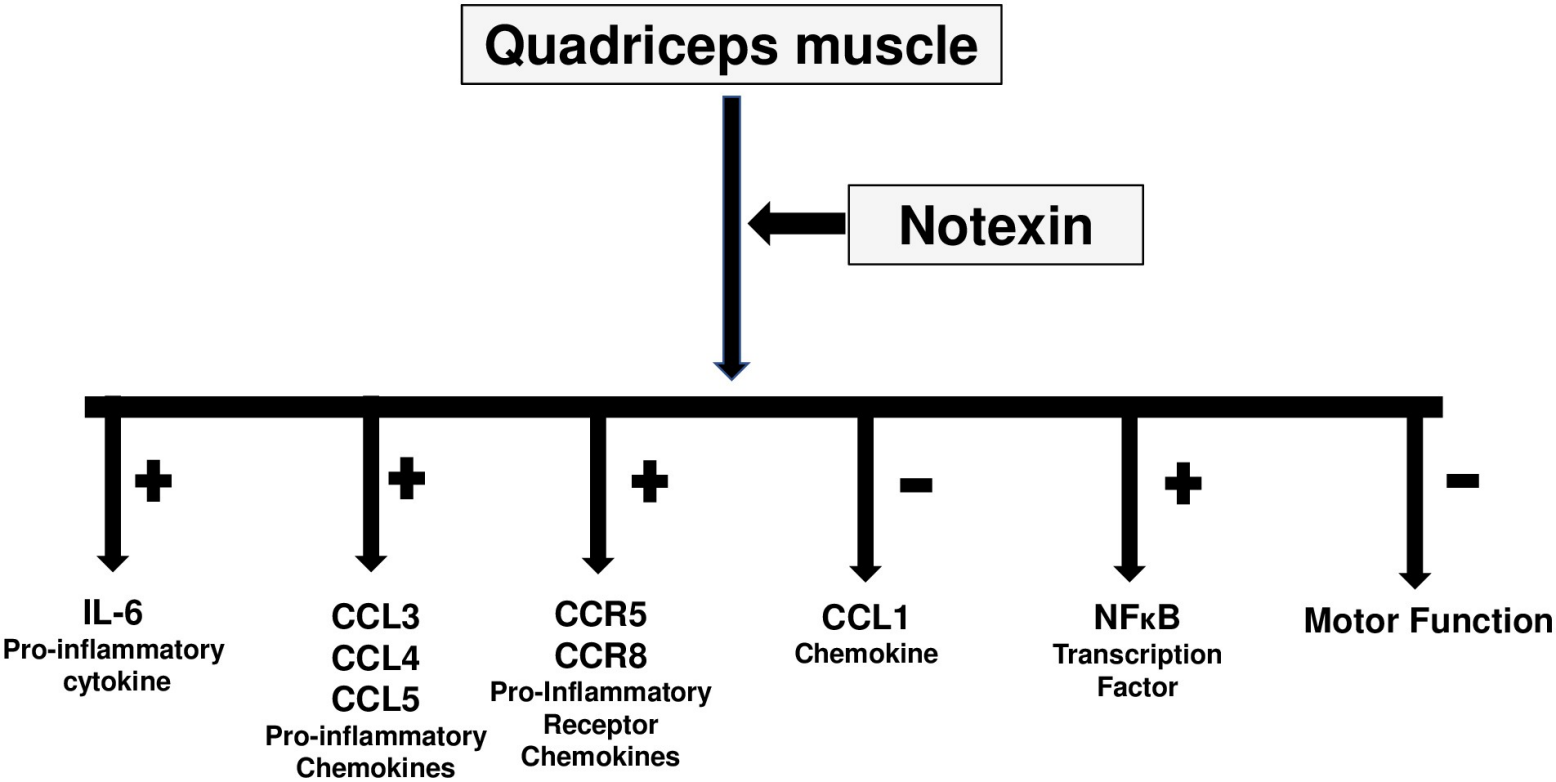
Notexin action on quadriceps muscle of rat. Increase in proinflammatory cytokines, chemokines, chemokines receptors and NFκB protein with decrease in CCL1 chemokine and motor function are indicated.

**Fig 8:**
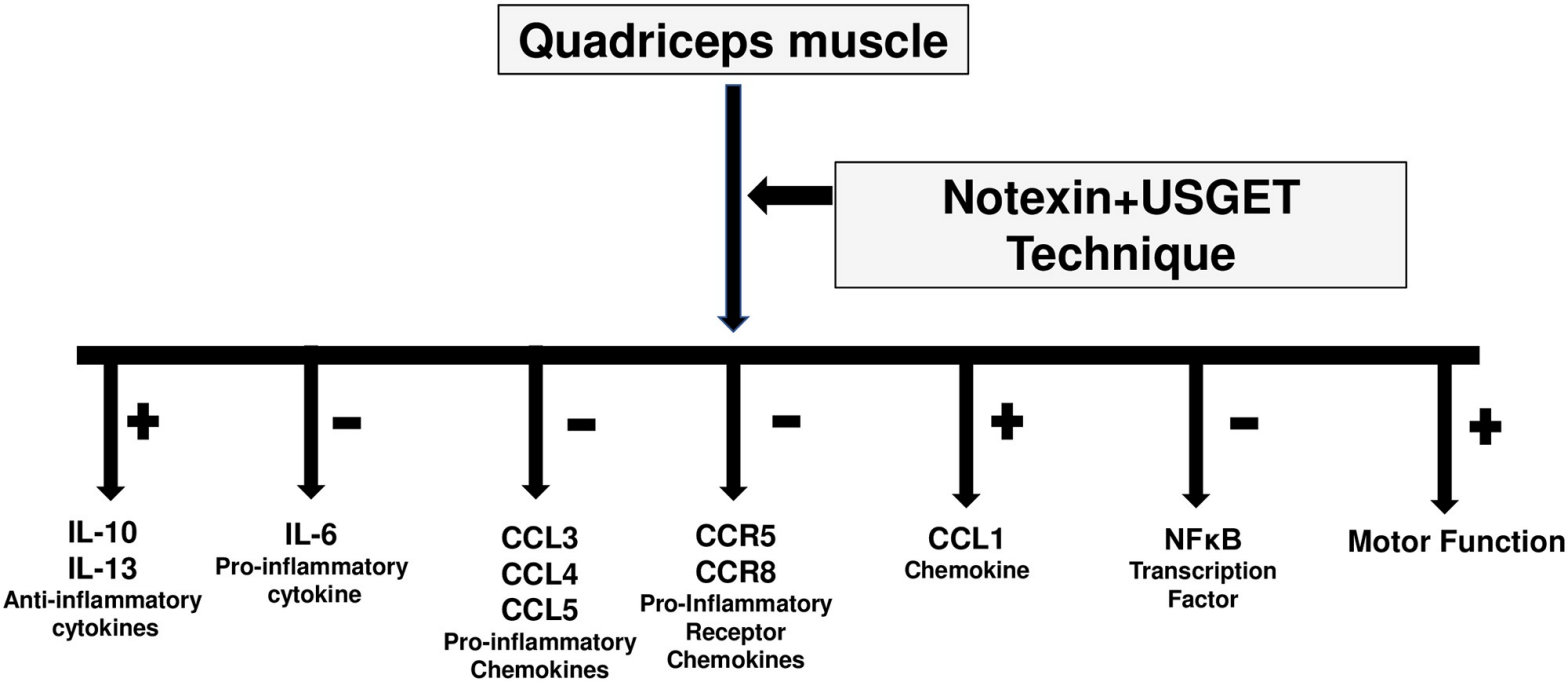
Notexin + USGTE (6 mA) technique action on quadriceps muscle of rat. Increase in anti-cytokines, CCL1 chemokine and motor function with decrease in proinflammatory cytokines, chemokines, chemokines receptors and NFκB protein are indicated.

All changes detected by the USGET technique used in inflammation have been corroborated by the recovery of motor functions detected in this study. To sum up, our study demonstrated an increase in inflammation and decreased motor function after notexin addition in the quadriceps muscle (Fig 7). Furthermore, significant improvements after USGET technique application were detected, with a decrease in pro-inflammatory proteins and an increase of anti-inflammatory mediators. Furthermore, all positive effects of USGET lead to a recovery of decreased motor function produced by notexin. This investigation explains the effectiveness of USGET treatment detected in humans (Fig 8). In any case, therapeutic interventions, such as percutaneous intra-tissue electrolysis and pulsed-dose radiofrequency, appear promising, but require further studies to confirm their efficacy.

In conclusion, our study demonstrates that the USGET technique prevents the increase of pro-inflammatory mediators, such as IL-6 cytokine and CCL3, CCL4 and CCL5 chemokines induced by notexin in the quadriceps muscle. Furthermore, USGET technique also showed a decrease in the CCR5 receptor of the chemokines mentioned above. Moreover, a decrease in CCR8 and NF-ᴋB expression was detected after USGET treatment. On the contrary, an increase in anti-inflammatory mediators (IL-10 and IL-13) after USGET treatment can recover from notexin-induced damage. These results would support the idea that USGET treatment could help muscle recovery after toxic damage justifying its use.

## Supporting information

S1 Raw images (PPTX)

## Abbreviations

CCL: Chemokine
IᴋB: Inhibitor kappa B
IL-1: Interleukine 1
NFᴋB: Nuclear Factor kappa B
NO: Nitric oxide
NOT: Notexin
ROS: Radical Oxygen Stress
TNF: Tumour Necrosis Factor
USGET: Ultrasound-guided electrolysis
