# Supplementary material for: Protective action of ultrasound-guided electrolysis technique on the muscle damage induced by notexin in rats
Source: PLoS One. 2022 Nov 28;17(11):e0276634. doi: 10.1371/journal.pone.0276634 (PMC9704622; doi:10.1371/journal.pone.0276634)
Supplement: S1 Raw images — (PPTX) [file pone.0276634.s001.pptx]

## Slide 1
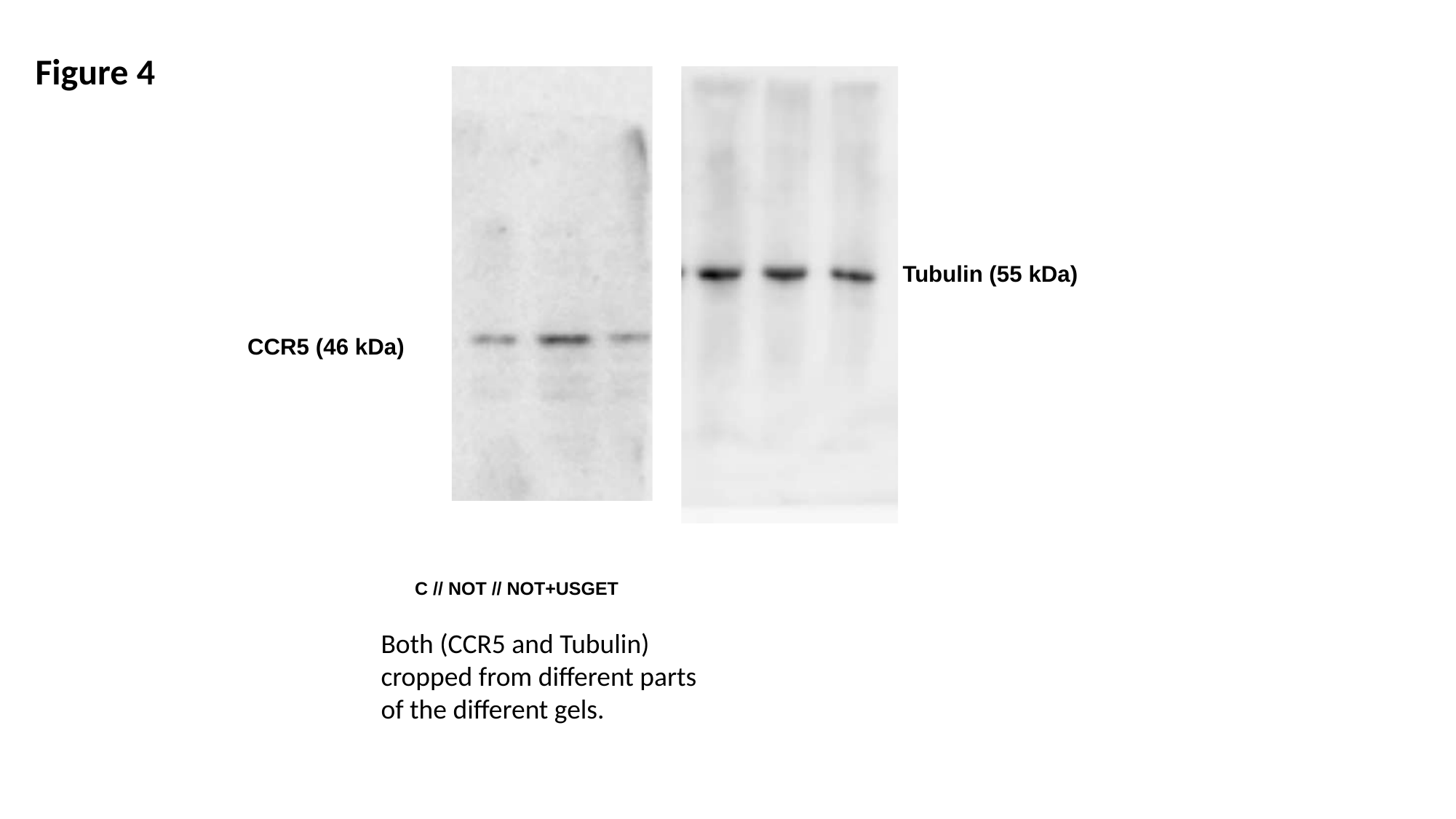

Figure 4
Tubulin (55 kDa)
CCR5 (46 kDa)
Both (CCR5 and Tubulin) cropped from different parts of the different gels.
 C // NOT // NOT+USGET

## Slide 2
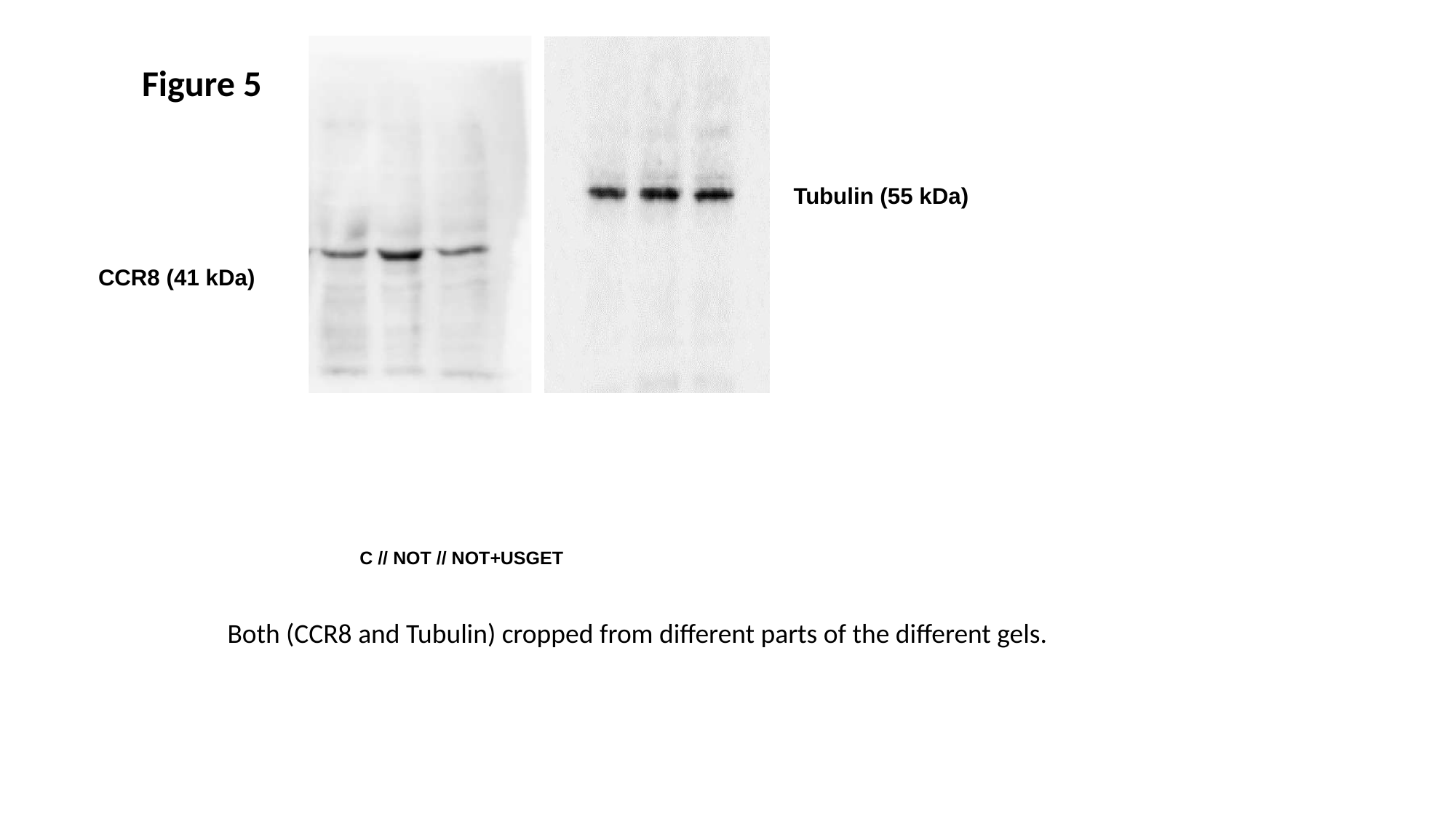

Figure 5
Tubulin (55 kDa)
CCR8 (41 kDa)
Both (CCR8 and Tubulin) cropped from different parts of the different gels.
 C // NOT // NOT+USGET

## Slide 3
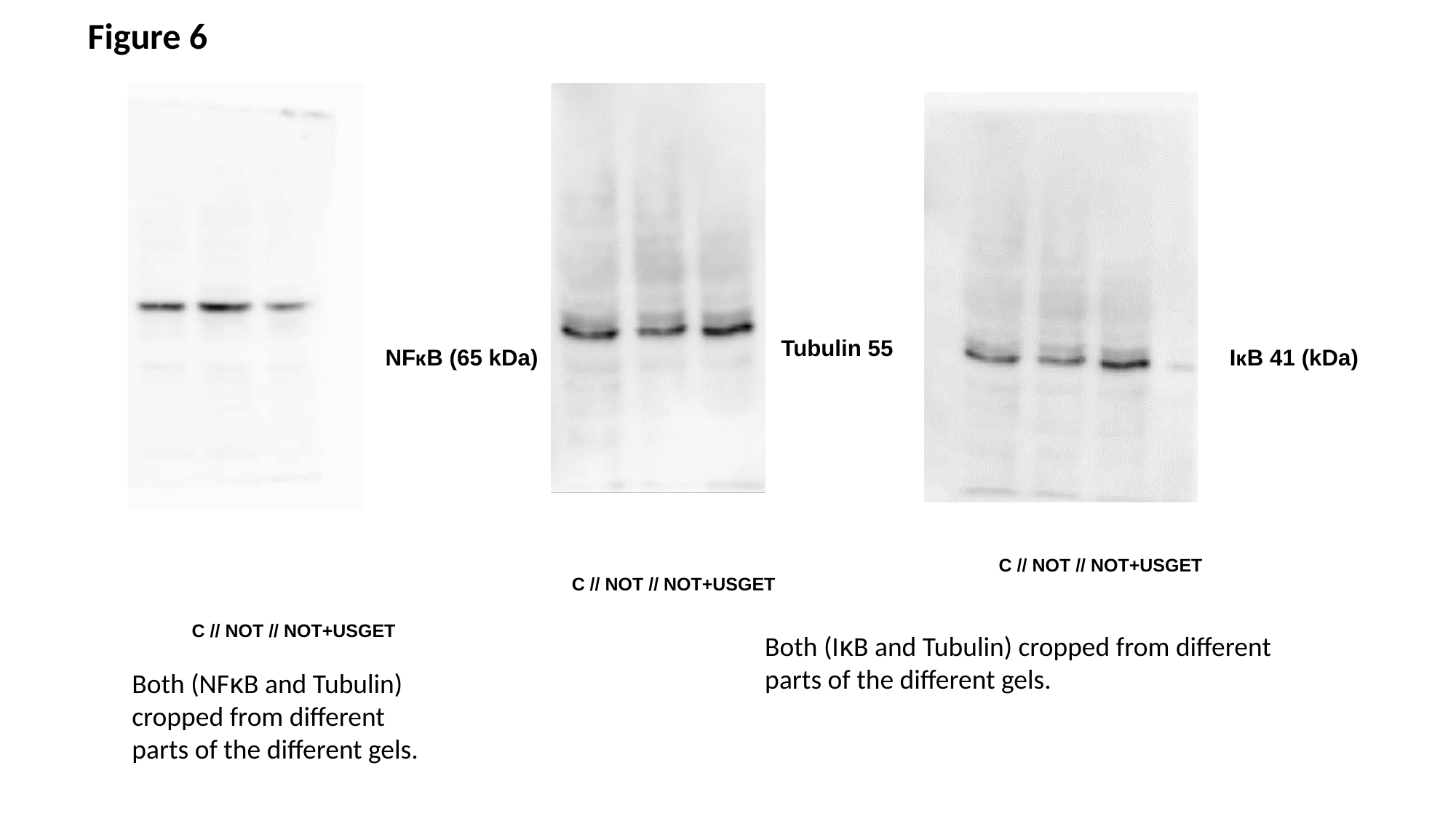

Figure 6
NFᴋB (65 kDa)
Both (NFκB and Tubulin) cropped from different parts of the different gels.
 C // NOT // NOT+USGET
Tubulin 55
IᴋB 41 (kDa)
Both (IκB and Tubulin) cropped from different parts of the different gels.
 C // NOT // NOT+USGET
 C // NOT // NOT+USGET
